# Effect of Intraocular Forward Scattering and Corneal Higher-Order Aberrations on Visual Acuity after Descemet’s Stripping Automated Endothelial Keratoplasty

**DOI:** 10.1371/journal.pone.0131110

**Published:** 2015-06-19

**Authors:** Kazutaka Kamiya, Hitomi Asato, Kimiya Shimizu, Hidenaga Kobashi, Akihito Igarashi

**Affiliations:** Department of Ophthalmology, University of Kitasato School of Medicine, Kanagawa, Japan; Sun Yat-sen University, CHINA

## Abstract

**Purpose:**

To assess the relationship of intraocular forward scattering and corneal higher-order aberrations (HOAs) with best spectacle corrected visual acuity (BSCVA) after Descemet’s stripping automated endothelial keratoplasty (DSAEK), and to compare these parameters between DSAEK and non-Descemet’s stripping automated endothelial keratoplasty (n-DSAEK) groups.

**Methods:**

This retrospective study enrolled thirty eyes of 30 consecutive patients who underwent standard DSAEK, and who underwent successful phacoemulsification with intraocular lens implantation before DSAEK. The mean age at the time of surgery was 71.7 ± 10.4 years. We quantitatively evaluated the objective scattering index (OSI) using the double-pass instrument (OQAS II, Visiometrics) and corneal HOAs using Hartmann-Shack aberrometry (KR-9000PW, Topcon) 3 months postoperatively.

**Results:**

The mean OSI, corneal HOAs, and logMAR BSCVA 3 months after DSAEK were 7.91 ± 3.58, 0.43 ± 0.27 μm, and 0.32 ± 0.25, respectively. We found a significant correlation between the OSI and logMAR BSCVA (Spearman correlation coefficient r=0.714, p<0.001), but no significant association between corneal HOAs and logMAR BSCVA 3 months postoperatively (r=0.209, p=0.267). We found no significant differences in any postoperative parameters between the DSAEK and n-DSAEK groups (p>0.05).

**Conclusions:**

Our pilot study demonstrated that the postoperative corrected visual acuity was significantly correlated with intraocular forward scattering, but not with corneal HOAs in post-DSAEK eyes, suggesting that intraocular forward scattering plays a more essential role in postoperative visual performance than corneal aberrations after DSAEK. The detailed visual performance, such as HOAs and intraocular scattering, after n-DSAEK appears to be essentially equivalent to that after DSAEK.

## Introduction

Descemet’s stripping automated endothelial keratoplasty (DSAEK) has been reported to be effective for the treatment of corneal endothelial dysfunction.[1–3] This surgical technique has several surgical advantages over penetrating keratoplasty (PK) in terms of faster visual rehabilitation, less surgically-induced astigmatism, less induction of graft rejection, and preservation of biomechanical properties. However, we do encounter some patients whose best spectacle-corrected visual acuity (BSCVA) was not very good, even when the transparency of the donor and recipient corneas appeared to be excellent after DSAEK in a clinical setting. This may be attributed to the induction of higher-order aberrations (HOAs) and/or light scattering by the cornea, especially by the anterior stroma and the interface between the donor and recipient corneas. Although several studies on corneal or ocular HOAs after DSAEK have so far been published,[4–15] there are only a few studies on both HOAs and intraocular scattering, which play a major role in visual performance in post-DSAEK eyes.[13,15]. All these previous studies on intraocular scattering have been focused on the backward scattering of the cornea,[13,15–17] except for one study on the forward scattering of the eye, which plays a more essential role in visual performance than backward scattering, after DSAEK.[18] However, the measurement of forward light scatter or retinal stray light used in their study is a subjective psychophysical test, which can introduce variability into the results. In addition, comparison of these optical parameters after DSAEK and non-Descemet’s stripping automated endothelial keratoplasty (n-DSAEK) has not so far been conducted. The purpose of the present study is twofold: to retrospectively assess the relationships of intraocular forward scattering and corneal HOAs with corrected visual acuity in post-DSAEK eyes, and to compare these optical parameters between the DSAEK and n-DSAEK groups.

## Materials and Methods

### Study Population

Thirty eyes of 30 consecutive patients (11 men and 19 women), who underwent standard DSAEK or n-DSAEK by the same surgeon, and who underwent successful phacoemulsification with intraocular lens (KS-1, STAAR Japan, Chiba, Japan) implantation before DSAEK, were included in this retrospective study. Any concomitant eye diseases such as macular atrophy, epiretinal membrane, vitreous opacification, uveitis, severe glaucoma, and trauma, were excluded from the study. Although two eyes had posterior capsular opacification, we performed a Nd-YAG capsulotomy in these eyes before enrollment. The sample size in this study offered 86% statistical power at the 5% level in order to detect a correlation of 0.5. The patient age at the time of surgery was 71.7 ± 10.4 years (mean age ± standard deviation (SD); range, 41 to 85 years). Written informed consent was obtained from all patients for the DSAEK surgery. This retrospective review of the data was approved by the Institutional Review Board at Kitasato University and followed the tenets of the Declaration of Helsinki. Our Institutional Review Board waived the requirement for informed consent for this retrospective study. Patient data was anonymized before access and/or analysis.

### Descemet’s Stripping Automated Endothelial Keratoplasty Procedures

We selected standard DSAEK or n-DSAEK, depending on the primary disease of patients. We performed standard DSAEK when the patients had a pathological Descemet’s membrane (guttae) that might interfere with vision, such as Fuchs dystrophy. Otherwise, we performed n-DSAEK. For standard DSAEK, Descemet’s stripping was performed within an 8.0-mm diameter, and the recipient’s endothelium and Descemet’s membrane were carefully removed with forceps. For n-DSAEK, Descemet’s stripping was not performed. Both standard DSAEK and n-DSAEK were performed through a 5.0-mm corneoscleral temporal incision after administering topical anesthesia. A precut donor was trephinated at an 8.0-mm size in diameter and was gently inserted into the anterior chamber using forceps and a Busin glide spatula. Air was carefully injected into the anterior chamber to unfold the graft. Ten minutes after the injection of the air, the air was partly replaced with balanced salt solution (BSS; Alcon, Fort Worth, TX). The corneoscleral tunnel incision was closed with three single or interrupted 10–0 nylon sutures. All subjects had inferior peripheral iridotomies and draining keratotomies during surgery. After surgery, steroidal (0.1% betamethasone, Rinderon, Shionogi, Osaka, Japan) and antibiotic (0.5% levofloxacin, Cravit, Santen, Osaka, Japan) medications were topically administered 4 times daily for 1 month, and then the frequency was steadily reduced.

### Assessment of Visual Acuity, Intraocular Scattering and Higher-order Aberrations

We assessed logMAR uncorrected visual acuity (UCVA), logMAR BSCVA, objective scattering index (OSI), corneal HOAs, corneal astigmatism, central corneal thickness, preoperatively and 3 months postoperatively, and corneal endothelial cell density 3 months postoperatively. Visual acuity measurement was performed using a Snellen chart with Japanese letters at a distance of 5 m with best correction (but not with habitual correction). The corneal astigmatism was measured using the autorefractometer (RK-5, Canon, Tokyo, Japan). The central corneal thickness were measured using an ultrasound pachymeter (DGH-500, DGH Technologies, Exton, US). The endothelial cell density was determined with a non-contact specular microscope (SP-8800, Konan, Nishinomiya, Japan). The manufacturer's software automatically produced an endothelial cell density measurement by visually comparing the cell size in the image with the predefined patterns on the screen.

The OSI was measured with Optical Quality Analysis System (Visiometrics, Terrassa, Spain) for a 4.0-mm pupil. The OSI is an objective evaluation of intraocular scattered light, and the index is calculated by evaluating the amount of light outside the double-pass retinal intensity point spread function image in relation to the amount of light on the center. In the particular case of the instrument OQAS, the central area selected was a circle of a radius of 1 minute of arc, while the peripheral zone was a ring set between 12 and 20 minutes of arc.[19] The OSI for normal eyes would range around 1, while values over 5 would represent highly scattered systems. The manifest refractive error of the subjects was fully corrected during these measurements; the spherical error was automatically corrected by the double-pass system, and the cylindrical error was corrected with an external lens, because the uncorrected refractive error directly affects the optical outcome of the system.

Corneal HOAs were measured with Hartmann-Shack aberrometry (KR-9000, Topcon, Tokyo, Japan) for a 4-mm pupil. The root-mean-square of the third-order Zernike coefficients was utilized to represent third-order aberrations, the root-mean-square of the fourth-order coefficient to represent fourth-order aberrations. Total HOAs were calculated as the root-mean-square of the third- and fourth-order coefficients. We performed at least 3 measurements in each device, and the averaged values were used for statistical analysis. The room illumination was kept low (approximately 25 lux) during testing. All examinations were performed by experienced optometrists.

### Statistical Analysis

All statistical analyses were performed using a commercially available statistical software (Ekuseru-Toukei 2010, Social Survey Research Information Co, Ltd., Tokyo, Japan). The normality of all data samples was first checked by the Kolmogorov-Smirnov test. Since the data did not fulfill the criteria for normal distribution, the Spearman correlation coefficient was calculated to assess the relationships of OSI and HOAs with logMAR BSCVA. The Mann-Whitney U test and the Fisher’s exact test were used to compare the data between the two subgroups. Unless otherwise indicated, the results are expressed as mean ± SD, a value of p<0.05 was considered statistically significant.

## Results

Preoperative patient demographics and diagnosis of the study population are summarized in Table 1. All surgical procedures were uneventful. No eyes were lost during 3-month follow-up in this series. The preoperative endothelial cell density obtained from the records of the eye banks that supplied the donor corneas was 2784 ± 243 cells/mm^2^ (range, 2353 to 3231 cells/mm^2^). Donor graft dislocation requiring an air re-injection and a transient pupillary air block requiring a partial release of air from the paracentesis were found in 3 eyes (10%) and 1 eye (3%), respectively. No other vision-threatening complications, such as iatrogenic primary graft failure and endothelial rejection, were seen at any time during the observation period.

**Table 1:** Preoperative demographics and diagnosis of the study population undergoing DSAEK.

Preoperative demographics			
No. of patients	30		
No. of eyes	30		
Age (years)	71.7 ± 10.4 (range, 41 to 85)		
Gender	Male: Female = 11: 19		
logMAR UCVA	1.15 ± 0.47 (range, 0.40 to 2.00)		
logMAR BSCVA	0.95 ± 0.51 (range, 0.22 to 2.00)		
Corneal astigmatism (D)	2.02 ± 2.09 (range, 0.00 to 9.50)		
	DSAEK group	n-DSAEK group	P value
No. of patients	10	20	-
No. of eyes	10	20	-
Primary disease	Fuchs’ dystrophy, 8 eyes	Post laser iridotomy, 13 eyes	
	Trauma, 1 eye	Post intraocular surgery, 6 eyes	
	Corneal endothelial dystrophy, 1 eye	Unknown origin, 1 eye	
Age (years)	72.8 ± 8.4 (range, 63 to 85)	71.1 ± 11.4 (range, 41 to 84)	1.000
Gender	Male: Female = 5: 5	Male: Female = 6: 14	0.292
logMAR UCVA	1.00 ± 0.48 (range, 0.40 to 1.70)	1.22 ± 0.46 (range, 0.52 to 2.00)	0.250
logMAR BSCVA	0.79 ± 0.47 (range, 0.40 to 1.70)	1.03 ± 0.52 (range, 0.22 to 2.00)	0.231
Corneal astigmatism (D)	2.12 ± 1.18 (range, 0.50 to 4.25)	1.97 ± 2.45 (range, 0.00 to 9.50)	0.209

DSAEK = Descemet’s stripping automated endothelial keratoplasty, logMAR = logarithm of the minimal angle of resolution, UCVA = uncorrected visual acuity, BSCVA = best spectacle corrected visual acuity, D = diopter.

**Fig 1:**
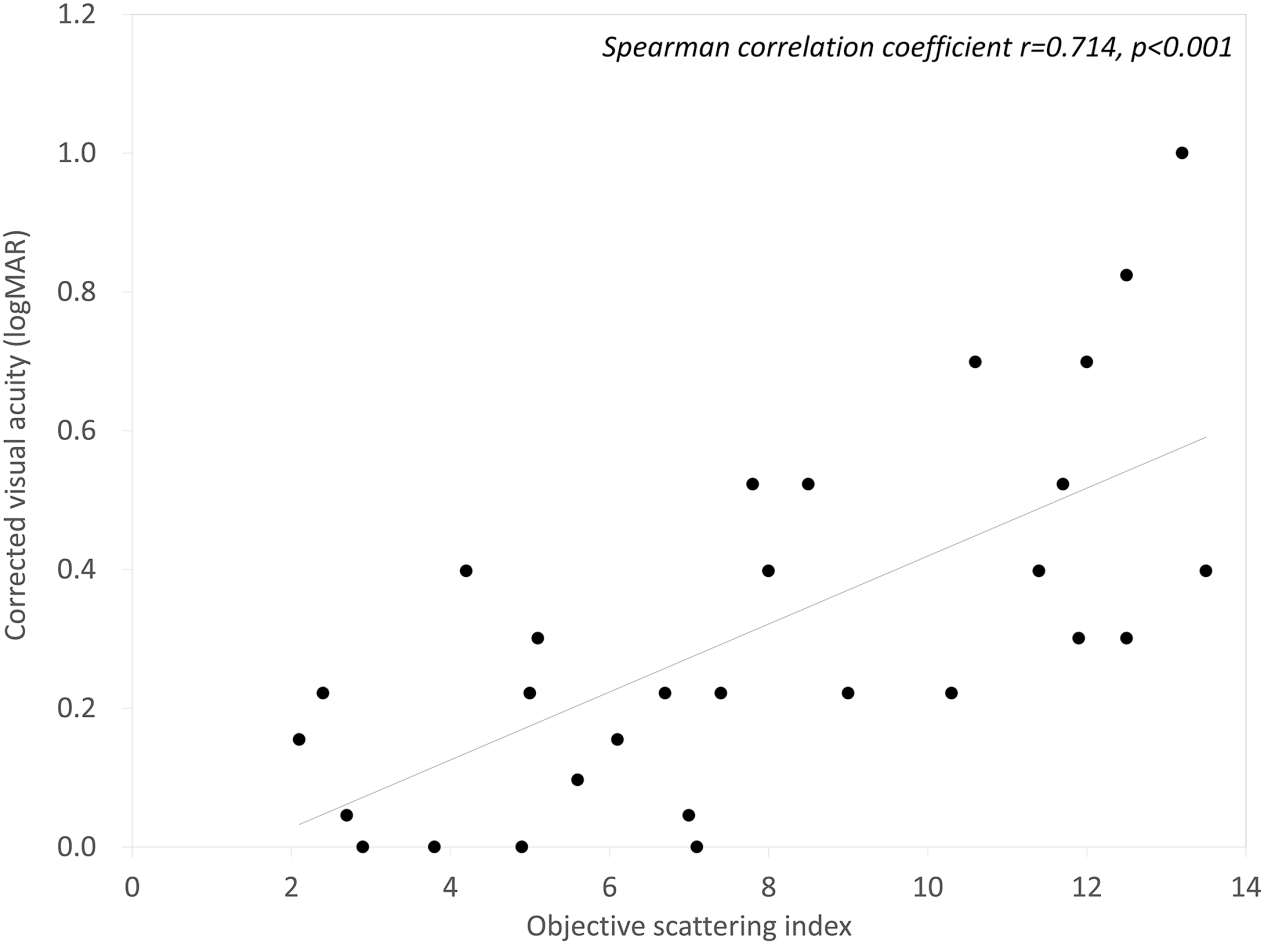
A graph showing a significant correlation between the objective scattering index (OSI) and best spectacle corrected visual acuity (BSCVA) (Spearman correlation coefficient r = 0.714, p<0.001).

**Fig 2:**
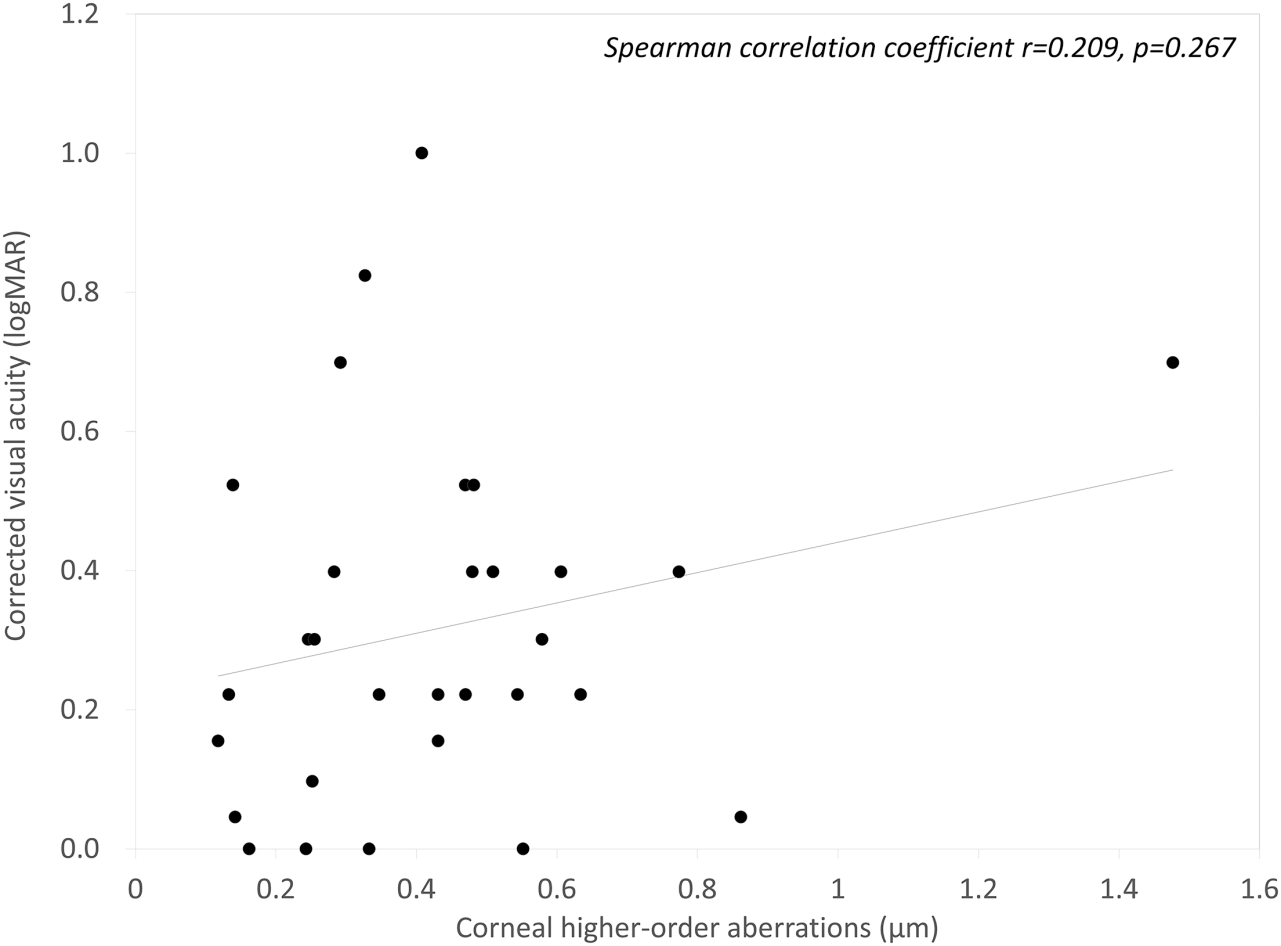
A graph showing no significant association between corneal higher-order aberrations and best spectacle corrected visual acuity (BSCVA) (Spearman correlation coefficient r = 0.209, p = 0.267).

Postoperative patient demographics are summarized in Table 2. The mean OSI, corneal HOAs, and logMAR BSCVA 3 months after DSAEK was 7.91 ± 3.58, 0.43 ± 0.27 μm, and 0.32 ± 0.25, respectively. We found a significant correlation between the OSI and logMAR BSCVA 3 months postoperatively (Spearman correlation coefficient r = 0.714, p<0.001)(Fig 1). On the other hand, we found no significant association between corneal HOAs and logMAR BSCVA 3 months postoperatively (r = 0.209, p = 0.267) (Fig 2).

**Table 2:** Postoperative demographics of the study population undergoing DSAEK.

Postoperative demographics			
No. of patients	20		
No. of eyes	20		
logMAR UCVA	0.68 ± 0.36 (range, 0.15 to 1.70)		
logMAR BSCVA	0.33 ± 0.26 (range, 0.00 to 1.00)		
OSI	8.11 ± 3.70 (range, 2.1 to 13.5)		
Corneal HOAs (μm)	0.48 ± 0.30 (range, 0.12 to 1.48)		
Corneal astigmatism (D)	2.84 ± 1.70 (range, 1.00 to 6.75)		
Central corneal thickness (μm)	618.5 ± 67.4 (range, 448 to 766)		
Endothelial cell density (cells/mm^2^)	1430 ± 364 (range, 838 to 2076)		
	DSAEK group	n-DSAEK group	P value
No. of patients	7	13	-
No. of eyes	7	13	-
Age (years)	70.9 ± 6.3 (range, 63 to 82)	70.3 ± 13.0 (range, 41 to 84)	0.603
Gender	Male: Female = 4: 3	Male: Female = 5: 8	0.642
logMAR UCVA	0.57 ± 0.27 (range, 0.30 to 1.05)	0.73 ± 0.38 (range, 0.15 to 1.70)	0.336
logMAR BSCVA	0.31 ± 0.30 (range, 0.00 to 0.80)	0.34 ± 0.24 (range, 0.00 to 1.00)	0.496
OSI	7.83 ± 3.27 (range, 2.1 to 12.5)	8.25 ± 3.90 (range, 2.4 to 13.5)	0.937
Corneal HOAs (μm)	0.57 ± 0.43 (range, 0.12 to 1.48)	0.43 ± 0.19 (range, 0.13 to 0.77)	0.905
Corneal astigmatism (D)	3.20 ± 2.23 (range, 1.00 to 6.75)	2.64 ± 1.29 (range, 1.25 to 5.50)	0.873
Central corneal thickness (μm)	615.3 ± 53.9 (range, 529 to 714)	620.2 ± 73.6 (range, 448 to 766)	0.721
Endothelial cell density (cells/mm^2^)	1612 ± 323 (range, 1073 to 2076)	1333 ± 347 (range, 838 to 1919)	0.122

logMAR = logarithm of the minimal angle of resolution, UCVA = uncorrected visual acuity, BSCVA = best spectacle corrected visual acuity, OSI = objective scattering index, HOAs = higher-order aberrations, D = diopter, DSAEK = Descemet’s stripping automated endothelial keratoplasty, n-DSAEK = non-Descemet’s stripping automated endothelial keratoplasty.

**Fig 3:**
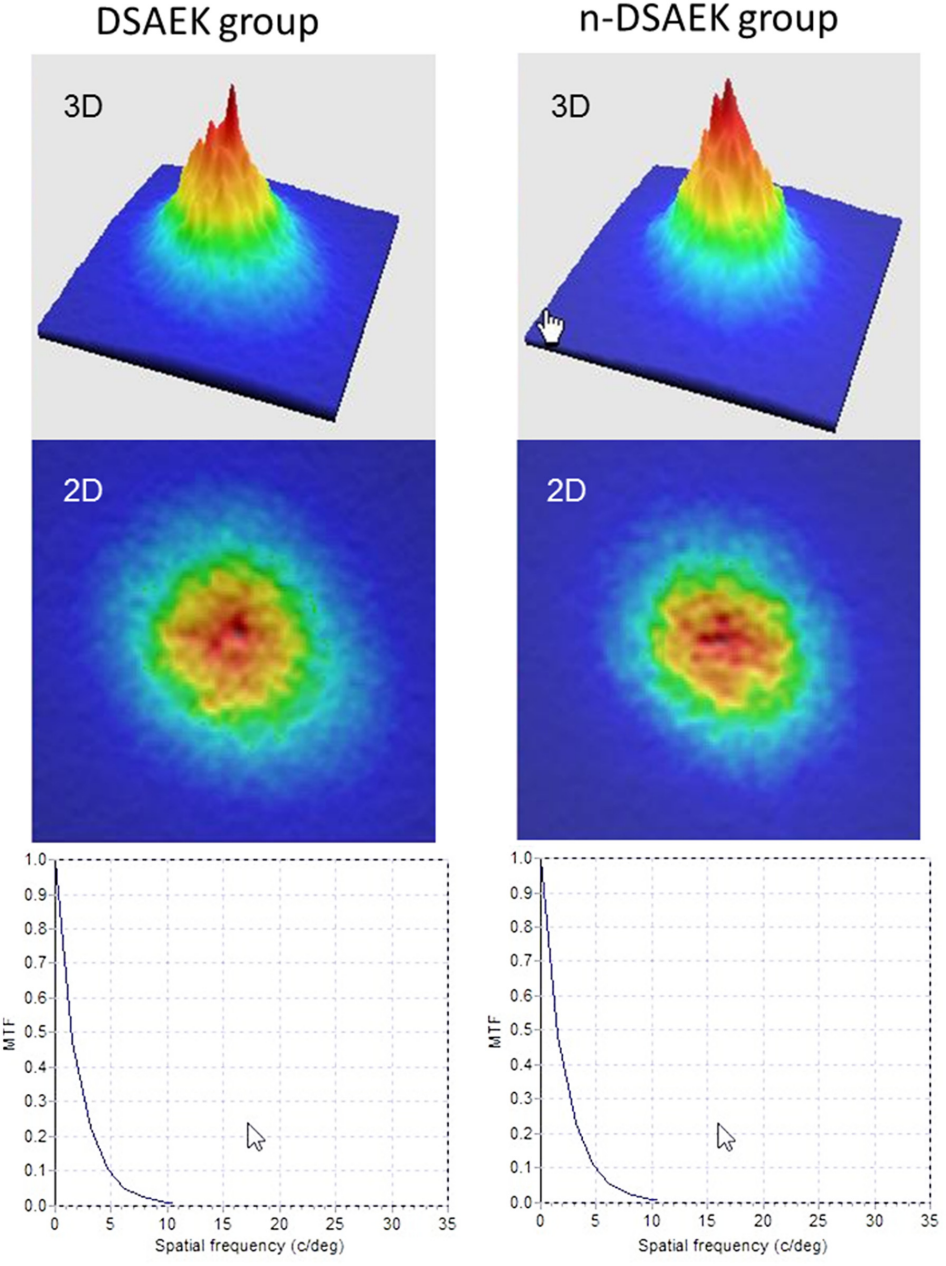
The double-pass images of eyes undergoing Descemet’s stripping automated endothelial keratoplasty (DSAEK) and non-Descemet’s stripping automated endothelial keratoplasty (n-DSAEK). MTF = modulation transfer function.

In the subgroup analysis, postoperative demographics of the DSAEK and n-DSAEK groups were listed in Table 2. Fig 3 shows representative examples of the double-pass images of eyes in the DSAEK and n-DSAEK groups. We found a significant correlation between the OSI and logMAR BSCVA 3 months postoperatively in the DSAEK group (Spearman correlation coefficient r = 0.644, p = 0.044) and in the n-DSAEK group (r = 0.666, p = 0.001), but no significant association between corneal HOAs and logMAR BSCVA 3 months postoperatively in the DSAEK group (r = 0.092, p = 0.800) or in the n-DSAEK group (r = 0.229, p = 0.331). We found no significant differences between the DSAEK and n-DSAEK groups in terms of OSI (Mann-Whitney U- test, p = 0.860), corneal HOAs (p = 0.509), logMAR UCVA (p = 0.930), logMAR BSCVA (p = 0.773), corneal astigmatism (p = 0.825), central corneal thickness (p = 0.538), or endothelial cell density (p = 0.692), 3 months postoperatively.

## Discussion

In the present study, our results demonstrated that the postoperative BSCVA was significantly correlated with the OSI, but not with corneal HOAs in post-DSAEK eyes, suggesting that intraocular forward scattering plays a more essential role in visual performance after DSAEK than corneal HOAs. As far as we can ascertain, this is the first study to objectively assess the forward scattering of the eye after DSAEK using the double-pass instrument. Although the exact etiology of this forward scattering remains unanswered, we assume that the scattering is largely derived from the interface haze between the donor and the recipient corneas and/or anterior stromal opacification of the cornea, since all eyes underwent successful IOL implantation and no concomitant eye diseases such as posterior capsular opacification were observed in this series.

There have been several studies performing detailed analysis of visual performance such as corneal or intraocular scattering and HOAs in post-DSAEK eyes.[4–18] Patel et al [18] were the first to report that BSCVA was significantly associated with intraocular forward scattering, but not with the backward light scattering by the cornea 6 months after DSAEK, suggesting the importance of intraocular forward scattering for predicting the postoperative visual performance in eyes undergoing DSAEK. Koh et al [13] showed a significant association between BSCVA and the backward scattering of the cornea after DSAEK using the rotating Scheimpflug camera. Hindman et al [15] recently demonstrated that the improvements in visual performance occurring over the first year after DSAEK were significantly associated with decreasing scattering of light, but that there were no significant changes in the ocular HOAs during this time. They [15] concluded that the factors that induced light scattering, other than tissue thickness or corneal edema, significantly impacted the visual improvements that occurred over time. Patel et al stated that the increased backscattering significantly correlated with decreased high- and low-contrast vision in PK-treated eyes,[20] and that BSCVA was significantly correlated with forward light scattering in eyes treated with deep lamellar endothelial keratoplasty (DLEK).[21] All these previous findings after keratoplasty were in line with our current results in that the intraocular scattering plays a more vital role in visual performance after DSAEK than HOAs, although most studies focused merely on the backward scatter of the cornea.[13,15–18]

With regard to anterior corneal HOAs, there are still controversies about HOAs on visual performance in patients undergoing DSAEK. Some studies demonstrated a significant correlation between BSCVA and anterior corneal HOAs,[4–7,11,12] whereas others demonstrated no significant correlation between them in such patients.[5,6,8–10,13,15] BSCVA was not also significantly associated with posterior corneal HOAs in any previous studies. Although the morphology of the posterior surface of the donor cornea may play some role in visual performance after DSAEK, we assume that the contribution of posterior corneal HOAs on visual acuity is smaller than anterior corneal HOAs. Further studies are still necessary to clarify this point.

In the subgroup analysis, our results also showed no significant differences in the postoperative OSI, corneal HOAs, logMAR UCVA, or logMAR BSCVA, between the DSAEK and n-DSAEK subgroups, suggesting that the presence or absence of Descemet’s membrane does not significantly affect visual acuity, higher-order aberrations, or intraocular forward scattering after surgery, possibly because this membrane is thin and is morphologically homogeneous. To our knowledge, this is also the first study to compare the detailed optical parameters after DSAEK and n-DSAEK. Although the primary disease and the surgical indication for n-DSAEK were different from that for DSAEK, the detailed visual performance, such as HOAs and intraocular scattering, both after n-DSAEK, seems to be essentially equivalent to that after DSAEK in a clinical setting.

There are several limitations to this study. Firstly, the relatively small amount of sample data and the fact that the design was retrospective. Although the sample size in this study offered > 90% statistical power at the 5% level, a prospective study with a large number of patients is required to confirm our preliminary findings. Secondly, the possible source of the intraocular forward scattering cannot be identified, although that of the backward scattering can be localized to the entire host cornea, not simply to the lamellar interface.[18] The origin of the backward scatter may be partially attributed to subepithelial fibrosis and ultrastructural changes in response to chronic dysfunction of the cornea. Patel et al [21] demonstrated that increased backscatter originated not only from the posterior cornea (interface) but also from the host cornea in post-DLEK eyes. However, the backward scattering did not appear to be a good indicator of visual performance in post-DASEK eyes.[18] We believe, on the basis of our preliminary data, that the objective assessment of intraocular forward scattering is clinically useful for predicting postoperative visual performance in eyes undergoing DSAEK. Thirdly, the maximum follow-up period was set at 3 postoperative months. A further long-term study is necessary to assess the relationship of BSCVA with the OSI and corneal HOAs in the late postoperative period.

In summary, our pilot study supports the view that BSCVA was significantly correlated with the forward scattering of the eye, but not with corneal HOAs in post-DSAEK eyes. It suggests that forward scattering of the eye plays a more important role in visual performance than corneal HOAs after DSAEK in a clinical setting. In addition, the detailed visual performance, such as HOAs and intraocular scattering, after n-DSAEK was essentially equivalent to that after DSAEK. Further long-term studies with a far greater number of subjects are required in order to confirm our preliminary findings.
